# Satisfaction with Access to Health Services: The Perspective of Estonian Patients with Rheumatoid Arthritis

**DOI:** 10.1100/2012/257569

**Published:** 2012-04-19

**Authors:** Kaja Põlluste, Riina Kallikorm, Kersti Meiesaar, Margus Lember

**Affiliations:** ^1^Department of Internal Medicine, University of Tartu, L. Puusepa 6, 51014 Tartu, Estonia; ^2^Department of Internal Medicine, Tartu University Hospital, L. Puusepa 6, 51014 Tartu, Estonia; ^3^Department of Public Health, University of Tartu, Ravila 19, 50411 Tartu, Estonia

## Abstract

In this cross-sectional study we explained the possible determinants of satisfaction with access to health services in patients with rheumatoid arthritis (RA). Of the 2000 randomly selected Estonian adult patients with RA, a total 1259 completed the survey. Regression analysis was used to analyse the predictors of patients' satisfaction with access to health services. Half of the respondents were satisfied with their access to health services. Factors that had a negative impact on satisfaction included pain intensity, longer waiting times to see the doctors, as well as low satisfaction with the doctors. Transportation costs to visit a rheumatologist and higher rehabilitation expenses also affected the degree of satisfaction. Patients who could choose the date and time at which they could visit the rheumatologist or who could visit their “own” doctor were more likely to be satisfied than patients whose appointment times were appointed by a healthcare provider.

## 1. Introduction

Rheumatoid arthritis (RA) is a chronic and progressive disease that has become an important cause of disability and morbidity and a drain on human and monetary resources. The prevalence of RA varies from 0.2 to 1.1 percent, with a higher prevalence in older age groups and women [1]. RA patients, like most chronically ill people, are dependent on health care services, and timely access to health services is an important issue. The results of previous studies have demonstrated that early diagnosis and early introduction of specific therapy was associated with a better disease outcome in RA [2, 3]. The rapid accessibility and availability of care are valued highly by the RA patients themselves [4, 5].

Accessibility has been defined as an important mark of quality in health care and could be defined as an absence of undue financial limits or limits of time or distance [6]. However, several studies have found that people with chronic conditions are less satisfied with the quality and access to health services than the other population [7, 8]. As frequent users of health services, patients with chronic conditions have more opportunities to experience difficulties in access, such as long waiting times, costs of care, fragmentation of care, and lack of continuity and coordination of care [7, 9–12]. The costs of care predicting the overall satisfaction with access to health services might be related to the patient's insurance status, copayments, and, for employed people, the opportunity cost of taking time to see a provider, which is measured by the loss of hourly wages [12]. Additionally, the satisfaction with access could be determined by organisational aspects, such as obtaining referrals, ease of arranging appointments, and the opportunity to be seen on the patient's day of choice [12–14]. Patients with RA expect their primary care doctors to provide quick referrals as well as rapid access to a rheumatologist following referral. They also expect that, if discomfort from RA increases, the health care provider would be available within a reasonable time [5].

Patients' satisfaction with accessibility has been found to be associated with a number of patient characteristics such as age, self-reported health status, and quality of life (QoL). In general, patients with better self-reported health status have rated their satisfaction with access to care better than those with poor health [5, 15]. Worsened functioning has been found to be associated with dissatisfaction with the ease and costs of care because patients with a poor health status have higher expectations and value shorter waiting times [16, 17]. It has been reported that patients with higher QoL evaluate chronic care management better [18], while patients with lower physical and mental health status scores were significantly less satisfied with the availability of care [19].

It is generally accepted that health systems need to be responsive to people's needs, which means it is important to handle special patient groups with unique needs in different ways [20]. In Estonia, one of the objectives of the health policy is to ensure that high-quality healthcare is available to all persons according to their needs [21]. The Estonian healthcare system has undergone several reforms since the early 1990s, starting with the implementation of social health insurance in 1992. Since then, health insurance coverage has not changed significantly and incorporates approximately 95 percent of the population. The new Health Insurance Act from 2002 brought in additional user fees for insured people (up to €1.60 per hospital day and up to €3.20 per visit to an outpatient specialist, while visits to family doctors (FDs) remain free for insured people). Furthermore, the patient's copayment of medical rehabilitation services and reimbursement of the costs of medicines was defined. In order to guarantee access to healthcare according to proven medical need, every healthcare provider in Estonia should have a waiting list. According to the 2006 and 2007 waiting list standards, FDs had to be able to see a patient with a chronic illness within three working days of an appointment request, while the waiting time for an outpatient specialist could not exceed four weeks after a referral [22].

Since the early 1990s, within the reorganized primary health care (PHC) system, PHC doctors have taken over a number of responsibilities that had previously fallen into the category of specialized outpatient care, including management of the care of chronically ill people. Currently, the care for chronically ill people (including patients with RA) in Estonia is shared between the two levels of the health care system—PHC and specialised care. The role of specialists in the management of chronic patients is mostly defined as a consultant and patients need referrals from their FDs [23–26]. A previous study found that, in 2003, rheumatic patients in Estonia made up 16.9 percent of all FDs' patients, while their number of visits constituted 33.5 percent of the total visits to a FD [27]. As demonstrated in earlier studies in Estonia, access to PHC services as well as specialists is good [23, 24, 27].

However, our 2005 study found that people with chronic conditions were less satisfied with their access to health services than the rest of the population. No significant differences were found between their waiting times to see a FD or a specialist, but more frequent visits to specialists, as well as difficulties seeing the specialist, predicted lower satisfaction with access to health services. On the other side, satisfaction with FDs and specialists predicted more expressed satisfaction with access [26]. It was also found previously that people who are satisfied with their doctor have more positive attitudes about the health reforms and evaluate the functioning of whole health system better [23, 25]. Thus, patient satisfaction with doctors might have an impact on the patients' evaluation of access as well and could, therefore, be considered as a possible predictor of satisfaction with access.

This current study focused on patients with RA as a specific group of chronically ill people in order to explain the possible determinants of satisfaction with access to health services. These determinants were patient characteristics, QoL, use of health services, financial, distance, time, and organisational aspects of the access, as well as satisfaction with doctors.

## 2. Methods

### 2.1. Patients

The study was conducted between October 2007 and January 2008 among adult Estonian patients with RA. Altogether, 2000 patients were randomly selected from the Estonian Health Insurance Fund (EHIF) database 2006. The total study population (*N* = 8814) included all persons who (1) had at least one point of contact with health services providers (FDs, rheumatologists, other out-patient specialists, hospital and rehabilitation services) in 2006 due to RA, (2) were at least 18 years old, and (3) had an RA diagnosis (M05 or M06 according to ICD-10). All patients included in the study sample were asked to provide informed written consent. The study design was approved by the Ethics Review Committee on Human Research of the University of Tartu. As the EHIF database does not include information about the patient's native language, all respondents received the questionnaire in Estonian and Russian. A reminder letter with a new questionnaire was sent to all nonrespondents four weeks later.

### 2.2. Questionnaire

This study is part of the RA patients' survey entitled “Quality of life: coping with illness and satisfaction with access to health services.” A questionnaire developed for this study included sociodemographic variables, health care utilisation, satisfaction with health care and providers of health services, use of medicines, adaption with disease and management of everyday life, information about the disease and sources of information, and self-reported direct costs of care for the patient. The patients' health status and quality of life (QoL) was measured by the 36-Item Short Form Health Survey (SF-36) developed at RAND as part of the Medical Outcomes Study, which is one of the most commonly used generic health status instruments [28]. The questionnaires were self-administered, consisting of multiple-choice questions, and patient's self-reported data and scales to measure the satisfaction and QoL.

The choice of questions for this survey was based on the analysis of the results of a qualitative study. In 2005-2006, six focus group interviews were conducted with 27 RA patients to explain their experiences about their health care [29]. Before the survey, the face and content validity of the questionnaire was tested in the Department of Internal Medicine of the Tartu University Clinics. Firstly, five RA patients with different backgrounds and disease histories completed the questionnaires without assistance and then discussed the results with the researcher. In order to estimate the reliability of the questions describing the patients' ratings, Cronbach's alpha was calculated for items describing the satisfaction with health services and service providers (0.70) and SF-36 instrument (0.95).

### 2.3. Study Variables

The variables included in this study were as follows. The *patient socioeconomic characteristics* were age, gender, education (primary, secondary, and university education), employment status as employed or not employed (retired, student, or unemployed), place of residence (urban area, rural area), personal status (either single or living together with another person(s)), and income. To explain the *patient's health status and Qol,* eight domains were calculated on the basis of data collected with SF-36 instrument. The* history of RA* was characterised by the duration of the disease (up to three years, 4–10 years, 11–20 years, 21 years, and more). *Comorbidity* was assessed using the question “Do you have any other permanent or chronic illness in addition to RA?”, with the answers “yes” and “no.” Information about the *use of health services* was collected with the questions “Have you, because of your RA, seen a doctor (FD or rheumatologist) during the past 12 months/been admitted in the hospital/received any medical rehabilitation services?”. Those respondents who answered “yes” were asked to indicate how many times they had seen a doctor and how many days they had spent in hospital. In order to estimate the access to health services, the following self-reported aspects were studied: (1) time factor (length of waiting time to see FD and rheumatologist), (2) distance factors (place of residence and time spent visiting the FD and rheumatologist), (3) financial factors (annual expenses for care, including visit fees, inpatient fees, costs for rehabilitation, and costs for transportation), (4) organisational factors related to the appointment time—respondents were asked whether their last visit to the rheumatologist was 1 = the first available time appointed by the healthcare provider or 2 = they had been able to choose the most suitable time and date for themselves), and (5) satisfaction with the FD and rheumatologist. The length of waiting time to see the FD was defined as follows: 1 = admitted on the same day as requested, 2 = admitted one or two days later, 3 = admitted three or four days later, 4 = admitted five to seven days later, and 5 = more than one week later. The length of waiting time to see the rheumatologist was as follows: 1 = up to one week, 2 = eight to 14 days, 3 = three to four weeks, and 4 = more than one month. The satisfaction with the FD and rheumatologist, as well as overall level of satisfaction with access to health care, were measured using the following five-point scale: 1 = satisfied, 2 = somewhat satisfied, 3 = somewhat dissatisfied, 4 = dissatisfied, 5 = do not know.

### 2.4. Statistical Analysis

Descriptive statistics and frequencies were computed for each item in the questionnaire. The items that were used as *independent variables* with ordinal and nominal values (e.g., background and health characteristics, use of and access to health services, satisfaction with the doctors) as well as continuous variables (e.g., age, income, dimensions of QoL, number of visits to the FD and rheumatologist, number of hospital days due to RA, time spent visiting the FD and rheumatologist, and patient's costs). The satisfaction with access to health services was determined as a *dependent variable*.

After the preliminary analysis of data, the dependent variable and items describing the satisfaction with doctors were dichotomised from the original five levels into two categories (1 = satisfied and somewhat satisfied, 2 = somewhat dissatisfied, dissatisfied, and do not know). The items describing the waiting times were dichotomised as well because of associations with the dependent variable: (1) waiting time to see the FD (1 = up to one week, 2 = more than one week) and rheumatologist (1 = up to one month, 2 = more than one month). The distribution and mean values of the independent variables were compared in two groups: patients who were satisfied with their access to health services and those who were not satisfied. The chi-square test was used to compare the distribution of variables with ordinal and nominal values, as well as to assess the representativeness of the sample. The ANOVA test was used to compare quantitative variables like age, QoL, time, and costs. The level of statistical significance was set at *P* < 0.05.

The predictors of satisfaction with access to health services were calculated using binary logistic regression. All items that were found to be significantly (*P* < 0.05) associated with the dependent variable were included in the model. The effect of independent variables on patient satisfaction with access to health services was expressed as odds ratios (ORs) with 95 percent confidence intervals. A difference was considered to be statistically significant if the *P* value was less than 0.05. All statistically nonsignificant variables were excluded from the final model. The data was analysed using SPSS 15.0 statistical software for Windows.

## 3. Results

### 3.1. Sample Description

Feedback was received from 1427 respondents (71.4 percent). Of these, 168 respondents dropped out of the study for the following reasons: the patient had moved to an unknown address or the patient had died, the patient was unable to respond due to his health status, the patient decided not to participate in the study, or did not complete the entire questionnaire. All these 168 respondents were excluded from the statistical analysis. The respondents who did not answer the question describing the satisfaction with access to health services (*n* = 22) were also excluded from this study. Thus, the final number of respondents included in this study was 1237 (a response rate of 62 percent). The nonrespondents were younger than the respondents (36.2% versus 21.5% were younger than 50 years, *P* < 0.0001), but the two groups did not differ in gender. The comparison of the structures of total study population, sample, respondents, and nonrespondents by gender and age is presented in detail elsewhere [30, 31].

Of the respondents, 17.6 percent were males and 82.4 percent were females. The age of the respondents ranged from 19 to 93 years (mean 59.2 ± 13.1). Seventy-nine percent of the respondents were older than 50 years of age. The mean duration of RA was 11.6 ± 11.5 years. Twenty-nine percent of the respondents were single, while 71 percent were married or lived with other family members. Sixty-two percent of the respondents lived in urban areas and 38 percent in rural areas.

The education distribution of the respondents showed that 21 percent had primary school education (up to nine years), 61 percent had secondary school education (10 or 12 years), and 18 percent had university education. Less than half of the respondents (43 percent) were employed, and 57 percent were retired or unemployed. The average monthly income was €279.40, while the median income was €223.60. The average amount of self-reported costs of care for RA patients per month was €19.50, which was seven percent of patients' average monthly income.

Half of the respondents were either satisfied (14 percent) or somewhat satisfied (36 percent) with their access to health services. More than one-third of the respondents were dissatisfied (28 percent were somewhat dissatisfied and 10 percent were not satisfied), and 12 percent of the respondents had no opinion on the matter. Satisfaction with their FD and rheumatologist was rather high: 86 percent and 85 percent of respondents were satisfied or somewhat satisfied with their doctors, respectively.  Table 1 shows the background and health characteristics of the patients according to their satisfaction with access to the health services. A significant association was found only between the respondent's employment status and satisfaction with access (*P* < 0.01).

Due to RA, most of the respondents had visited their FDs and rheumatologist during the last 12 months (74 percent and 73 percent, resp.), 25 percent of the respondents received medical rehabilitation services, and 20 percent were admitted to hospital (for an average of 9.7 days).  Table 2 indicates the associations between satisfaction with access to health services and characteristics related to healthcare use. The percentage of patients using various outpatient and inpatient health services, as well as the self-reported numbers of visits to FD and rheumatologist, were the same for satisfied patients as they were for patients who were not satisfied with their access to health services, although patients who spent more days in hospital were more satisfied with access to care (*P* < 0.01). For the last time visiting the rheumatologist for 47 percent of respondents, the time was appointed by the healthcare provider as the first available time; they also were less satisfied with their access to the health services (*P* < 0.01). Fifty-three percent of the respondents who visited their rheumatologist reported that this was their first opportunity to see their “own” doctor at a suitable date and time; that is, the appointment time was chosen by the patient. Patients who spent less time visiting their FD or rheumatologist were more satisfied with their access to health services (*P* < 0.05). Stronger associations (*P* < 0.0001) were found between waiting times for the doctors and satisfaction with access to health services. Patients who reported higher personal expenses for care (€267.40 versus €200.70, *P* < 0.01) were more likely to be less satisfied with their access to health services. In terms of the different kind of expenses, lower satisfaction with access to health services was significantly associated with higher fees for outpatient visits (€17.10 versus €13.70, *P* < 0.05), higher expenses related to rehabilitation services (€116.80 versus €69.60, *P* < 0.05), and higher transportation costs to see the rheumatologist (€7.30 versus €5.90, *P* < 0.05).

Eight dimensions were used to describe respondents' QoL. All of these dimensions were evaluated as being lower by those respondents who were less satisfied with access to health services. Despite this, statistically significant differences were found for only three dimensions: emotional well-being (*P* < 0.05), social functioning (*P* < 0.01), and pain (*P* < 0.0001) (see Table 3).

### 3.2. Predictors of Satisfaction with Access to Health Services

When analysing the coinfluence of sociodemographic variables, dimensions of QoL, satisfaction with doctors, as well as time, distance, financial, and organisational factors of access, five groups of determinants were found for predicting satisfaction with access to health services. These were (1) pain as health determinant, (2) time-related determinants (waiting time to see the doctor), (3) the organisational aspect of access as the opportunity for a patient to see their “own” doctor at the most suitable time and date, (4) financial determinants (transportation costs to see the rheumatologist and costs for rehabilitation services), and (5) satisfaction with doctors (Table 4). Longer waiting times to see the FD and rheumatologist and lower satisfaction with the FD and rheumatologist, as well as more severe pain, all had a negative impact on the level of satisfaction with access to health services. The low level of satisfaction was also a result of higher transportation costs and higher expenses for rehabilitation. Patients who were able to choose the date and time at which they would visit their rheumatologist or their “own” doctor were more likely to be satisfied with their access to healthcare than patients who were not able to choose the appointment time that was most suitable for them (i.e., whose appointment time was appointed by healthcare provider). None of the patient-related socioeconomic variables nor the use of healthcare was found to predict satisfaction with access.

## 4. Discussion

This study investigated the determinants of satisfaction with access to health services in the opinion of a specific group of chronically ill people: patients with RA. Previous studies had indicated that people with chronic conditions are less satisfied with access to health services than the remaining population, and the main predictors have been described as follows: availability and ease of getting to doctors, followup of patients, frequency of visits to doctors, costs of care, and patient characteristics and health conditions as well as satisfaction with the health system and doctors [5, 7–9, 12–15, 17–19, 26].

### 4.1. Health-Related Determinants of Satisfaction with Access

A number of studies have reported the positive associations between the satisfaction with healthcare accessibility and patient's health status and QoL [5, 15–19]. RA as a chronic and progressive disease affects not only the patients' general health status but also their quality of life. For that reason, instead of a single question of health status, the study used the SF-36 instrument to evaluate the different domains of QoL and their possible effect on satisfaction with access to healthcare. In PHC, for example, both the physical and mental component scores were associated with satisfaction with the availability of care [19]. The present study found that three of the eight dimensions of QoL were independently associated with the satisfaction of access–pain was one domain that described the physical component of QoL and there were two domains describing the mental health (emotional well-being and social functioning). Still, only pain, out of these three domains, was found to predict the satisfaction with access. As a domain of QoL, bodily pain captures the frequency of pain and the extent of interference with normal activities due to pain [28]. Pain is one of the most frustrating symptoms of RA and the most common motivation for seeking medical help. In case of pain, quick access to health services is essential [32]. This could be a major reason why pain has a more expressed effect on the satisfaction with access than other components of QoL. RA patients also prioritised availability of medical care in case that the discomfort from RA increases [5].

### 4.2. Time-Related Determinants of Access

Waiting time to see doctors has often been reported in the context of problems related to access to health services [7, 9, 10, 16]. For patients with RA, the timely access is associated with better outcomes, and RA patients themselves also rated rapid access to care as one of the most important priorities [2–5]. The current study also found that waiting time to see a rheumatologist or FD predicted satisfaction with access. Compared to our previous studies, the waiting time to see an FD has been more or less on the same level throughout the years [23, 24]. Although access to specialists in Estonia is better than that in some other countries [10, 33, 34], the waiting time to see a rheumatologist reported by study patients was longer than that reported in a 2004 study. In that study, the time required to receive a specialist appointment did not exceed three weeks, and almost half of the patients assessed the availability of rheumatologists as excellent [27]. This study suggested that people generally accept the standards of waiting times fixed in the contracts concluded between the health care providers and EHIF; for the FD, however, even a longer than standard [22] waiting time was acceptable. It has been argued that the top priority for PHC patients was to be seen on a day of their choice rather than to be seen quickly [14]. Thus, it is possible that some respondents whose waiting time was longer than three days chose the appointment day themselves, which means it did not affect their satisfaction with access. Still, if the waiting time to see the doctors exceeds the agreed standards, it can have a significant negative impact on satisfaction with access.

### 4.3. Choice of an Appointment Time as a Predictor of Satisfaction with Access to Health Services

In addition to the waiting times, the opportunity to choose an appointment time was found to be an essential determinant of satisfaction with access. In order to be satisfied with their access to health services, the ease of arranging appointments and the question of whether the patient could make an appointment on the day of their choice play an important role [13, 14]. There could be two reasons for this. Firstly, having appointments with the same doctor promotes better continuity of care, which is an essential factor in order for people with chronic conditions to be satisfied with access [10, 11]. Secondly, the impact that the opportunity to choose the appointment time has on their satisfaction with access might be related to the opportunity costs for the patient. This is primarily important for employed people who may lose some wages because they have to take time to see the doctor during their working hours [12].

### 4.4. Financial Determinants of Access

The cost of care has also been found to be a reason for dissatisfaction with access to care [9, 12]. This study found that higher costs for transportation to see a rheumatologist and costs for rehabilitation did affect the level of satisfaction. The geographical distribution of FDs' offices is more or less homogenous across the country, but most rheumatologists work in two bigger centres. This means that patients living outside these centres have to spend more money for transportation, which leads to lower satisfaction with access.

In general, the average proportion of direct costs of patient's income was rather low, and the total amount of costs did not affect the satisfaction with access. However, patients who used rehabilitation services also spent more money for co-payment, which had a negative impact on satisfaction with access. Rehabilitation is an essential part of RA management, but only one-quarter of respondents reported the use of rehabilitation services. Since 2002, however, the amount of rehabilitation services fully paid for by health insurance has been rather limited. In addition, the co-payment for the rehabilitation can be quite high [22]. The negative impact of higher expenses on satisfaction with access, as well as the low proportion of patients who received the rehabilitation, refers to financial barriers that restrict access to rehabilitation care.

### 4.5. Satisfaction with Doctors as a Predictor of Satisfaction with Access to Health Services

The study found that, in addition to the factors discussed above, the satisfaction with access to health services was also predicted by the patient's satisfaction with their FD and their rheumatologist. This result was similar to that of our previous study [26]. Generally speaking, there is very limited evidence about the impact of a patient's satisfaction on their satisfaction with access to health services. However, several studies have confirmed the high importance that healthcare consumers attach to interaction factors and the quality of the patient-practitioner relationship in general when they are evaluating care [15]. In RA patients' followup, the FDs and rheumatologists both have a significant role. As reported previously, patients with RA usually expect their FDs not only to have medical help available but also to provide a quick referral to a rheumatologist if the patient experiences increased discomfort due to RA [5]. If the patient's problems are already being successfully managed at the PHC level or if they receive a referral and appropriate help from the rheumatologist, they are usually satisfied with their doctors. Based on this understanding, the finding that patient satisfaction with doctors determines the satisfaction with access seems rather logical. However, this is a cross-sectional study, and the causality can move in both directions. It is possible that higher satisfaction with doctors is the result of accessible healthcare; this is a question for future research.

### 4.6. Strengths and Limitations of the Study

The main strength of this study is its data source. The Estonian Health Insurance Fund has a complete database of all insured people, which covers approximately 95 percent of the population. The study sample, which included more than one-fifth of all patients with RA who had contact with health services during the year, was representative of the total study population. The response rate is acceptable and comparable with other surveys [35]. However, younger respondents (up to 50 years) were underrepresented in the study and people aged 50 and over were slightly overrepresented. Nonresponse has been associated in different studies with both older and younger patients [35]. It is possible that, due to this response bias, the ratings of younger respondents could be less presented than those of older respondents. However, respondents' age was not found to predict satisfaction with access to health services nor was it associated independently with satisfaction rates. Another limitation is related to the risk of a recall bias given that respondents were asked to remember their utilisation within the previous twelve months. The question about the objectivity of patients' self-reporting about the use of health services has been raised in other studies as well [7]. Furthermore, the level of ratings may be related to the patient's personality and emotions at the moment of filling the questionnaire, which cannot be controlled. Still, the results of the present study were very similar to our previous results in terms of the main predictors of satisfaction with access to health services [26].

## 5. Conclusions

The results of our study demonstrated that about half of the Estonian RA patients are satisfied with their access to health services. The satisfaction with access was associated with some aspects of QoL, whereby greater expressed pain predicted a lower rate of satisfaction. Essential determinants of higher satisfaction with access were acceptable waiting times and the opportunity for a patient to see their doctor at a convenient time or access to rehabilitation services without financial limits. In addition, the satisfaction with one's FD and rheumatologist played a significant role in people's satisfaction with their access to health services.

## Figures and Tables

**Table 1:** Comparison of background and health characteristics in patients with rheumatoid arthritis according to their satisfaction with access to health services.

	Patients satisfied or somewhat satisfied with access to health services (*n* = 614)	Patients not satisfied with access to health services (*n* = 623)
Age group		
18–29	2.1	3.2
30–39	4.9	6.3
40–49	11.4	15.6
50–59	27.5	28.4
60–69	29.3	24.4
70 and older	24.8	22.2
Gender		
Male	18.9	15.9
Female	81.1	84.1
Place of residence		
Urban	63.0	60.4
Rural	37.0	39.6
Employment status*		
Employed	38.8	48.5
Not employed (retired, student, or unemployed)	61.2	51.5
Personal status		
Single	29.7	26.6
Living together with another person(s)	70.3	73.4
Duration of RA		
Up to three years	28.2	34.0
Four to ten years	31.1	27.2
11–20 years	21.5	20.7
21+ years	19.2	18.1
Comorbidity		
Yes	62.8	64.3
No	37.2	35.7

*Statistically significant difference between the groups that are satisfied and not satisfied with access to health services (*P* < 0.01) using a chi-square test.

**Table 2:** Comparison of use of health services and amount of contacts due to RA, expenses, waiting times, and options to make an appointment time to see the rheumatologist in patients with rheumatoid arthritis according to their satisfaction with access to health services.

	Patients satisfied or rather satisfied with access to health services (*n* = 614)	Patients not satisfied with access to health services (*n* = 623)
Percentage of respondents who, during the last 12 months due to RA, have		
Visited the FD (%)	73.5	76.7
Visited the rheumatologist (%)	74.5	73.5
Received medical rehabilitation services (%)	23.7	26.9
Been admitted in the hospital (%)	21.9	19.2
Average number of visits per year per person (mean ± SE)		
Family doctor	4.6 ± 0.2	4.7 ± 0.2
Rheumatologist	3.7 ± 0.1	3.4 ± 0.1
Average number of days spent in hospital per year per person (mean ± SE)**	11.1 ± 0.9	7.9 ± 0.6
Average time spent seeing the doctor (in minutes, mean ± SE)		
Family doctor*	101.4 ± 2.8	109.6 ± 3.0
Rheumatologist*	193.5 ± 6.4	224.0 ± 10.6
Self-reported average expenses for care per person (EUR, mean ± SE )		
Total expenses (per year)**	200.7 ± 13.3	267.4 ± 11.9
Visit fee (per year)*	13.7 ± 1.1	17.1 ± 1.2
Inpatient fees (per year)	14.1 ± 1.3	12.9 ± 1.3
Costs for rehabilitation (per year)*	69.6 ± 8.7	116.8 ± 17.7
Transportation costs to see the family doctor (per visit)	2.4 ± 0.1	2.6 ± 0.2
Transportation costs to see the rheumatologist (per visit)*	5.9 ± 0.3	7.3 ± 0.4
Waiting time to see the family doctor, %***		
Up to one week	96.2	88.3
More than one week	3.8	11.7
Waiting time to see the rheumatologist, %***		
Up to four weeks	76.2	56.5
More than four weeks	23.8	43.5
Last visit to the rheumatologist was (%)**		
First available time appointed by the healthcare provider	41.8	51.5
Appointment time chosen by patient	58.2	48.5

*P < 0.05; **P < 0.01; ***P < 0.0001, statistically significant differences between groups that are satisfied and not satisfied with access to health services (*P* < 0.01) using a chi-square test.

**Table 3:** Scores of dimensions of quality of life (mean ± SE) and satisfaction with access to health services in patients with rheumatoid arthritis.

	Patients satisfied or rather satisfied with access to health services (*n* = 614)	Patients not satisfied with access to health services (*n* = 623)
General health	31.74 ± 0.77	30.76 ± 0.76
Physical functioning	48.68 ± 1.14	47.92 ± 1.18
Role limitations due to physical health	29.10 ± 1.75	27.95 ± 1.70
Emotional wellbeing*	56.98 ± 0.90	53.77 ± 0.89
Role limitations due to emotional problems	36.82 ± 1.93	34.11 ± 1.84
Social functioning**	56.81 ± 1.17	52.07 ± 1.17
Vitality (energy/fatigue)	36.51 ± 0.79	34.95 ± 0.80
Pain***	41.10 ± 1.06	36.22 ± 1.02

*P < 0.05;   **P < 0.01;  ***P < 0.001, statistically significant differences between groups that are satisfied and not satisfied with access to health services using an ANOVA test.

**Table 4:** Determinants having an impact on the satisfaction with access to health services in patients with rheumatoid arthritis (Nagelkerke *R* ^2^ = 0.26).

	Odds ratio (OR)	95% CI for OR
Health-related determinants		
Pain (decrease by one point of score)	0.990	0.980–1.000
Time-related determinants		
Waiting time to see the FD		
Up to one week	1.000	
More than one week	0.767	0.652–0.902
Waiting time to see the rheumatologist		
Up to four weeks	1.000	
More than four weeks	0.760	0.674–0.856
Determinants related to the appointment time		
Last visit to the rheumatologist was		
First available time appointed by the healthcare provider	1.000	
Appointment time chosen by patient	1.470	1.057–2.049
Financial determinants		
Transportation costs related to the visit to rheumatologist (increase by one Euro)	0.965	0.933–0.998
Costs for rehabilitation (increase by one Euro)	0.997	0.994–0.999
Determinants related to the satisfaction with doctors		
Satisfaction with the FD		
Satisfied	1.000	
Not satisfied	0.350	0.197–0.617
Satisfaction with the rheumatologist		
Satisfied	1.000	
Not satisfied	0.342	0.201–0.582
